# Rathke Cleft Cyst Presenting as Panhypopituitarism in an Adolescent

**DOI:** 10.1210/jcemcr/luaf129

**Published:** 2025-06-10

**Authors:** Tarannum Bano, Najat Nasser Alghamdi, Abdulrahman Jalal Altwijri

**Affiliations:** Adult Endocrinology Department, King Saud Medical City, Riyadh 12746, Kingdom of Saudi Arabia; Adult Endocrinology Department, King Saud Medical City, Riyadh 12746, Kingdom of Saudi Arabia; Adult Endocrinology Department, King Saud Medical City, Riyadh 12746, Kingdom of Saudi Arabia

**Keywords:** panhypopituitarism, Rathke cleft cyst, short stature, puberty delay, hormone replacement therapy

## Abstract

Rathke cleft cyst (RCC) is a benign lesion of the pituitary region, more commonly diagnosed in adults, in whom it typically presents with headaches, visual disturbances, or endocrine dysfunction. In pediatric patients, RCC is rare and even less frequently associated with panhypopituitarism. We report a case of a 16-year-old male patient who presented with short stature and delayed puberty. Hormonal evaluation revealed deficiencies in cortisol, thyroid hormone, gonadotropins, testosterone, and growth hormone. Magnetic resonance imaging (MRI) of the pituitary showed a 10 × 12.5 × 11.3-mm RCC displacing the pituitary stalk. Hormone replacement therapy with hydrocortisone, levothyroxine, and growth hormone was initiated. After 12 months, the patient showed a 6-cm increase in height and mild regression of cyst size. This case highlights the importance of considering RCC in the differential diagnosis of adolescents with growth failure or delayed puberty, even in the absence of typical symptoms such as headache or visual impairment. Hormone replacement therapy can be a safe and effective first-line treatment for RCC-associated hypopituitarism in the absence of severe neurological symptoms. Surgery should be reserved for cases with substantial headache or vision-related complications. Regular clinical and radiological follow-up is essential to monitor hormonal function and cyst progression.

## Introduction

Rathke cleft cyst (RCC) is a benign lesion that occurs in the sellar and suprasellar regions, originating from remnants of the Rathke pouch. The peak incidence of RCCs is reported between ages 30 and 50 years, with a female predominance. Most RCCs are asymptomatic and may be discovered incidentally. Autopsy studies have reported their prevalence to be approximately 12% to 33% in adults [1]. In contrast, RCCs in children are rarer and often asymptomatic [2]. However, symptoms may arise due to compression of surrounding structures [3]. Regarding the growth of asymptomatic RCCs, one study observed that over a median follow-up period of 36 months, only 24.3% of patients experienced cyst enlargement, with 5.1% showing an increase greater than 3 mm. This suggests that the majority of RCCs remain stable over time [4]. The most common clinical symptoms of RCC include headaches, visual disturbances, and pituitary hormone deficiencies [5-7]. The clinical presentation, imaging findings, and surgical outcomes of pediatric RCC closely resemble those seen in adults. However, RCC-induced hypopituitarism in children has rarely been reported in the literature [8-10]. Additionally, cases of precocious puberty associated with RCC have been documented, highlighting the diverse endocrine manifestations of this condition [11]. We report the case of an adolescent boy with a large Rathke cleft cyst, resulting in panhypopituitarism, but without typical symptoms such as headache or visual disturbances. Our patient was managed conservatively, and follow-up showed mild regression in cyst size.

## Case Presentation

A 16-year-old boy was referred to the endocrinology clinic with complaints of short stature and delayed puberty, first noticed 2 years prior. He had been started on recombinant growth hormone therapy at a dose of 1 mg daily (25 mcg/kg/day) 3 months before presenting to our clinic. He was born of a full-term normal delivery, with no significant neonatal or childhood medical history. There was no history of birth injury, and his developmental milestones were normal. There was no family history of endocrine disorders. The patient denied symptoms such as headaches, visual disturbances, polyuria, polydipsia, weight changes, or hearing impairment.

## Diagnostic Assessment

**Figure 1.**
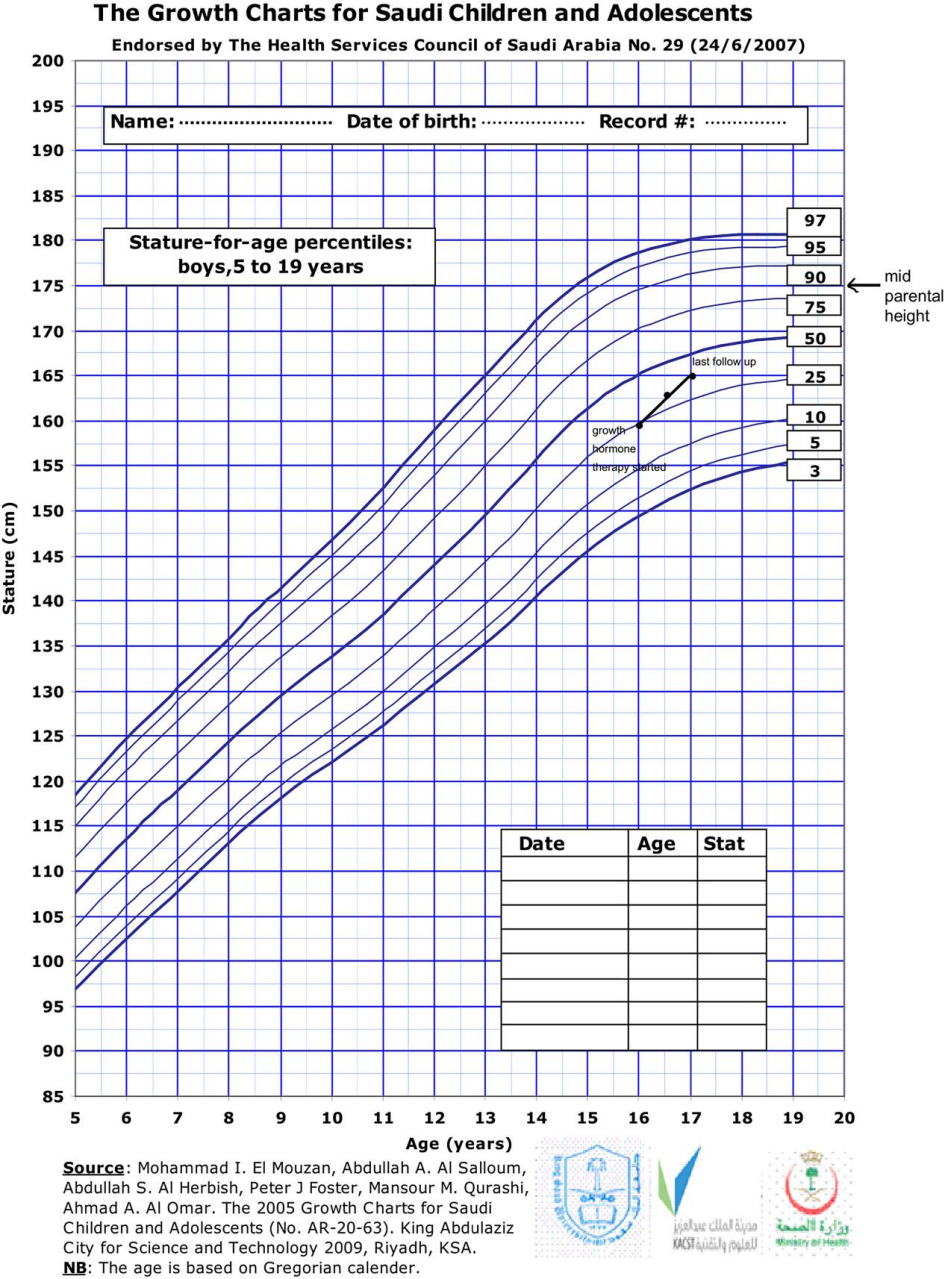
Growth chart; illustrating height progression over 1 year following the initiation of growth hormone therapy.

On examination, the patient's height was 161 cm, arm span 162 cm, and weight 40 kg. There was no documented height velocity available prior to presentation at our clinic. However, before initiating growth hormone therapy, his height was recorded as 159 cm (between the 10th and 25th percentiles on the growth chart for Saudi children and adolescents as shown in Fig. 1). The mother's height was 167 cm and the father's height 170 cm, giving a calculated mid-parental height of 175 cm. The patient’s systolic blood pressure was 90 mm Hg (<3rd percentile) and diastolic pressure was 60 mm Hg (<50th percentile). There was an absence of axillary and pubic hair, and bilateral testicular volume was 3 mL, consistent with Tanner stage 1. Bone age, assessed using the Greulich and Pyle method, was delayed and corresponded to 14 years.

Laboratory testing revealed significant deficiencies across multiple pituitary axes. The morning serum cortisol level was 50.86 nmol/L (1.84 µg/dL) (normal reference range, 102-535 nmol/L; 3.70-19.40 µg/dL**)**. Free thyroxine (FT4) was 5.46 pmol/L (0.42 ng/dL) **(**normal reference range: 12-22 pmol/L; 0.93-1.70 ng/dL**)**. The morning total testosterone level was 0.24 nmol/L (6.9 ng/dL) (normal reference range: 1.63-34 nmol/L; 47.01-980.62 ng/dL). Luteinizing hormone (LH) was 1.45 IU/L (normal reference range, 0.57-12.07 IU/L), while follicle-stimulating hormone (FSH) was 1.99 IU/L (normal reference range, 1.5-12.4 IU/L), serum prolactin level was 52 µg/L (1106 μIU/mL) (normal reference range, 3.46-19.40 μg/L; 73.61-404.25 μIU/mL**)**. Insulin-like growth factor-1 (IGF-1) was significantly reduced at 57 ng/mL (7.452 nmol/L) (normal reference range, 119-511 ng/mL; 15.55-66.80 nmol/L).

**Figure 2.**
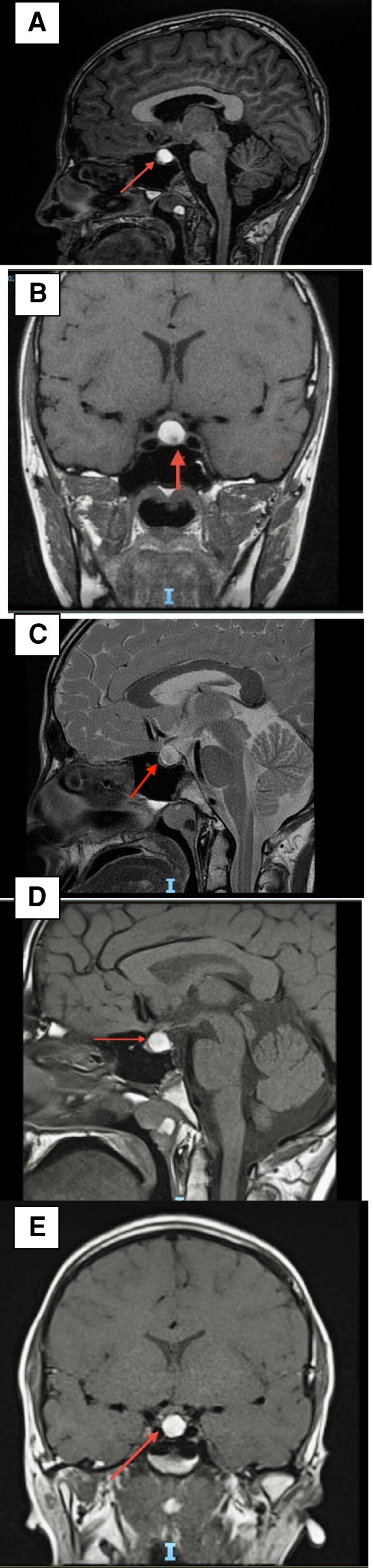
T1-weighted and T2-weighted magnetic resonance imaging showing a large sellar lesion exhibiting high signal intensity both on T1- and T2-weighted images, measuring approximately 10 × 12.5 × 11.3 mm in the anteroposterior, transverse, and craniocaudal dimensions, respectively. The lesion displaces the pituitary stalk superiorly and mildly abuts the optic chiasm. A, T1-weighted, sagittal view; B, T1-weighted, coronal view; and C, T2-weighted image. At 12 months’ follow-up there is a marginal interval decrease in the size of the pituitary lesion, measuring 1 × 1 × 1 cm (craniocaudal, anteroposterior, and transverse dimensions, respectively). D, T1-weighted, sagittal view; and E, T1-weighted, coronal view.

Magnetic resonance imaging (MRI) of the pituitary revealed a large, well-defined cystic sellar lesion exhibiting high signal intensity both on T1- and T2-weighted images. The lesion measured approximately 10 × 12.5 × 11.3 mm in the anteroposterior, transverse, and craniocaudal dimensions, respectively. It displaced the pituitary stalk superiorly and mildly abutted the optic chiasm. No enhancement was observed post contrast (Fig. 2A-2C). The findings were consistent with a large RCC. However, the differential diagnosis for cystic sellar lesions includes cystic pituitary adenoma, craniopharyngioma, arachnoid cyst, epidermoid cyst, and teratoma. Cystic pituitary adenomas typically demonstrate fluid-fluid levels, hemorrhagic debris, and septations on MRI. Craniopharyngiomas often contain calcifications and solid enhancing components. Arachnoid cysts, epidermoid cysts, and teratomas are more commonly located in the suprasellar cistern rather than the sellar region, which supports RCC as the most likely diagnosis in this case.

## Treatment

The patient was initiated on hormone replacement therapy with glucocorticoids, receiving hydrocortisone 10 mg in the morning and 5 mg in the afternoon. Levothyroxine 75 mcg once daily was started 48 hours after the initiation of hydrocortisone. Recombinant growth hormone therapy was subsequently titrated based on IGF-1 levels.

## Outcome and Follow up

Over 12 months of follow-up, the patient reported improved energy levels and experienced a total height gain of 6 cm, with 4 cm occurring within the first 6 months. Laboratory results demonstrated significant hormonal improvements: FT4 levels increased from 5.46 pmol/L (0.42 ng/dL) to 11.68 pmol/L (0.90 ng/dL) (normal reference range, 12-22 pmol/L; 0.93-1.70 ng/dL), and IGF-1 levels rose from 7.45 nmol/L (57 ng/mL) to 21.83 nmol/L (167 ng/mL) (normal reference range, 119-511 ng/mL; 15.55-66.80 nmol/L) (Table 1). No new visual or neurological symptoms were reported. For pubertal induction, low-dose testosterone therapy (50 mg intramuscular injection once a month) was initiated. Follow-up MRI revealed a marginal decrease in the size of the RCC, measuring approximately 1 × 1 × 1 cm in craniocaudal, anteroposterior, and transverse dimensions, respectively (Fig. 2D and 2E).

**Table 1.** Laboratory tests

Parameters	Result SI units (conventional units)		Normal range SI units (conventional units)
	On presentation	At 12-mo follow-up	
**FT4**	5.46 pmol/L (0.42 ng/dL)	11.68 pmol/L (0.90 ng/dL)	12-22 pmol/L (0.93-1.70 ng/dL)
**TSH**	2.71 mIU/L	1.49 mIU/L	0.27-4.2 mIU/L
**Testosterone**	0.24 nmol/L (6.9 ng/dL)	0.34 nmol/L (9.80 ng/dL)	1.63-34 nmol/L (47.01-980.62 ng/dL)
**LH**	1.45 IU/L	0.32 IU/L	0.57-12.07 IU/L
**FSH**	1.99 IU/L	1.26 IU/L	1.5-12.4 IU/L
**Prolactin**	52 μg/L (1106 μIU/mL)	21 μg/L (446.80 μIU/mL)	3.46-19.40 μg/L (73.61-404.25 μIU/mL)
**8 Am serum cortisol**	50.86 nmol/L (1.84 µg/dL)	—	102-535 nmol/L (3.70-19.40 µg/dL)
**IGF-1**	7.45 nmol/L (57 ng/mL)	21.83 nmol/L (167 ng/mL)	15.55-66.80 nmol/L (119-511 ng/mL)
**Serum sodium**	139 mmol/L	140 mmol/L	136-145 mmol/L

Abbreviations: FSH, follicle-stimulating hormone; FT4, free thyroxine; IGF-1, insulin-like growth factor; LH, luteinizing hormone; TSH, thyrotropin.

## Discussion

RCCs are benign, epithelium-lined cystic lesions that arise from remnants of the Rathke pouch. The clinical manifestation of pediatric RCC with panhypopituitarism is an infrequent occurrence [2]. Although the optimal management of pediatric RCCs remains uncertain, the available literature suggests the treatment decisions are primarily guided by the severity of symptoms [3, 12, 13]. Symptomatic RCCs, particularly those causing visual impairment or persistent headaches, often require surgery [14-16]. Our case report highlights an unusual presentation of RCC in an adolescent manifesting as isolated hypopituitarism without the typical symptoms of headache or visual disturbances. Given the absence of these common indications for surgery, we opted for conservative management with hormone replacement therapy, which is consistent with the current literature. Over a period of 12 months, the patient gained 6 cm in height, despite an interruption of growth hormone therapy for 3 months at the time of presentation to our clinic. Repeat imaging revealed a slight decrease in the cyst size, further supporting the role of conservative management in mildly symptomatic RCC cases such as isolated hypopituitarism. A comparable case reported by Bizzarri et al [17] described a case of a 14-year-old adolescent with RCC-related headache and hypopituitarism. Although cyst resection resulted in headache resolution, the patient continued to experience persistent hypothyroidism and diabetes insipidus at a 4-year follow-up. This underscores the potential risks of surgery, including new or persistent pituitary dysfunction, cyst recurrence, and other surgery-related complications [3, 8, 18]. A comparative analysis of surgery vs conservative management in 24 pediatric RCC cases demonstrated that many patients under conservative management experienced resolution of headaches, and 35% had cyst regression during follow-up [19].

The findings in our case reinforce the importance of individualized management of pediatric RCC. While surgery remains the standard of treatment for cases with significant visual or neurological symptoms, conservative management with close endocrine monitoring remains a viable option for those with isolated endocrine disturbances. Long-term follow-up, including repeat imaging and hormonal assessment, is crucial to detect any disease progression and ensure optimal patient outcomes.

## Learning Points

- RCC should be considered in the differential diagnosis of adolescents presenting with short stature or delayed puberty, even in the absence of headache or visual symptoms.
- Hormone replacement therapy is an effective first-line treatment for patients with RCC-induced hypopituitarism who do not exhibit severe neurological symptoms. Hydrocortisone should be initiated at least 24 hours before starting T4 replacement.
- Surgery should be reserved for cases with substantial headache or visual disturbances.
- Regular monitoring of pituitary function and follow-up imaging are essential to assess cyst progression and determine the continued need for hormone replacement therapy.

## Contributors

All authors contributed individually to the manuscript. N.A. diagnosed and managed the patient, A.A. managed the patient and reviewed the manuscript, and T.B. examined the patient file, wrote the case report, and submitted the manuscript. All authors reviewed and approved the final draft.

## Data Availability

Original data generated and analyzed for this case report are included in this published article.

## Abbreviations

FSH: follicle-stimulating hormone
FT4: free thyroxine
IGF-1: insulin-like growth factor
LH: luteinizing hormone
MRI: magnetic resonance imaging
RCC: Rathke cleft cyst; TSH, thyrotropin
